# *N*,*N*′-Diisopropyl-3,6-dimethoxy­naphthalene-2,7-disulfonamide

**DOI:** 10.1107/S1600536808021211

**Published:** 2008-07-16

**Authors:** Zu-Wei Song, Zhi-Qiang Hu

**Affiliations:** a College of Science, Qingdao Agricultural University, Qingdao 266109, People’s Republic of China; b College of Chemistry and Molecular Engineering, Qingdao University of Science and Technology, Qingdao 266042, People’s Republic of China

## Abstract

In the title compound, C_18_H_26_N_2_O_6_S_2_, all bond lengths and angles are normal. The crystal structure is stabilized by inter­molecular N—H⋯O hydrogen bonds.

## Related literature

For the crystal structures of related compounds, see: Henschel *et al.* (1996 ▶). For details of the biological activities of fluorine-containing compounds, see: Kamoshita *et al.* (1987 ▶). For catalytic activity, see: Zhang *et al.* (2007 ▶). For bond-length data, see: Allen *et al.* (1987 ▶).
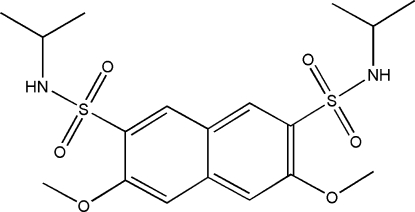


## Experimental

### 

#### Crystal data

C_18_H_26_N_2_O_6_S_2_
*M* *_r_* = 430.53
Monoclinic, 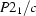

*a* = 17.229 (3) Å
*b* = 7.2532 (15) Å
*c* = 18.035 (4) Å
β = 108.35 (3)°
*V* = 2139.2 (8) Å^3^
*Z* = 4
Mo *K*α radiation
μ = 0.28 mm^−1^
*T* = 173 (2) K
0.50 × 0.38 × 0.22 mm

#### Data collection

Rigaku R-AXIS RAPID IP area-detector diffractometer
Absorption correction: multi-scan (*ABSCOR*; Higashi, 1995 ▶) *T*_min_ = 0.871, *T*_max_ = 0.940
8740 measured reflections
4889 independent reflections
4227 reflections with *I* > 2σ(*I*)
*R*_int_ = 0.020

#### Refinement

*R*[*F* ^2^ > 2σ(*F* ^2^)] = 0.046
*wR*(*F* ^2^) = 0.117
*S* = 1.16
4889 reflections
253 parameters
H-atom parameters constrained
Δρ_max_ = 0.36 e Å^−3^
Δρ_min_ = −0.49 e Å^−3^

### 

Data collection: *RAPID-AUTO* (Rigaku, 2004 ▶); cell refinement: *RAPID-AUTO*; data reduction: *RAPID-AUTO*; program(s) used to solve structure: *SHELXTL* (Sheldrick, 2008 ▶); program(s) used to refine structure: *SHELXTL*; molecular graphics: *SHELXTL*; software used to prepare material for publication: *SHELXTL*.

## Supplementary Material

Crystal structure: contains datablocks I, global. DOI: 10.1107/S1600536808021211/hg2423sup1.cif

Structure factors: contains datablocks I. DOI: 10.1107/S1600536808021211/hg2423Isup2.hkl

Additional supplementary materials:  crystallographic information; 3D view; checkCIF report

## Figures and Tables

**Table 1:** Hydrogen-bond geometry (Å, °)

*D*—H⋯*A*	*D*—H	H⋯*A*	*D*⋯*A*	*D*—H⋯*A*
N1—H1*A*⋯O2^i^	0.88	2.00	2.821 (2)	154
N2—H2*B*⋯O5^ii^	0.88	2.22	3.001 (2)	148
N2—H2*B*⋯O6^ii^	0.88	2.53	3.235 (2)	138

Symmetry codes: (i) 
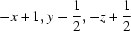
; (ii) 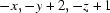.

**Fig. 1.**
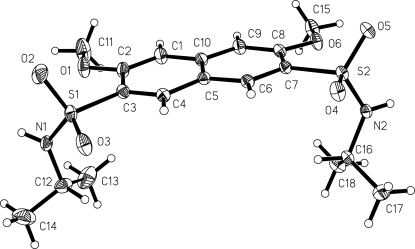
The molecular structure of (I), with atom labels and 40% probability displacement ellipsoids for non-H atoms.

**Fig. 2.**
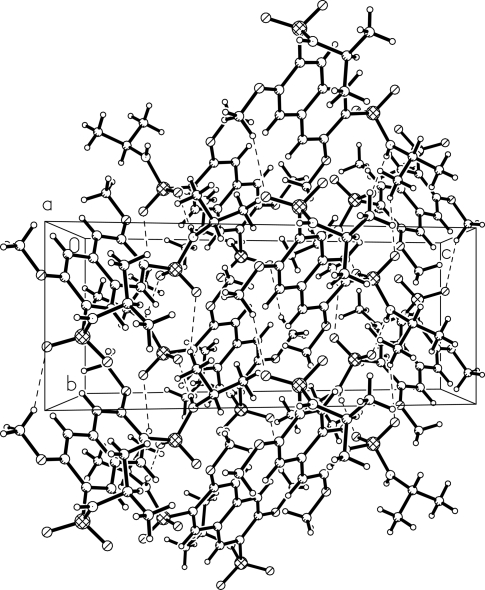
The packing of (I), viewed down the a axis, showing one layer of molecules connected by N—H···O hydrogen bonds (dashed lines).

## supplementary crystallographic information

### Comment

The sulfonamides form an important group in organic chemistry with many compounds containing sulfonamide groups possessing a broad spectrum of biological activities and can be widely used as herbicides (Kamoshita *et al.*, 1987). In addition, some compounds containing sulfonimide groups can be used as catalysts (Zhang *et al.*, 2007). Here, we report the crystal structure of the title compound, (I).

In (I) (Fig. 1), all bond lengths are normal (Allen *et al.*, 1987) and in good agreement with those reported previously (Henschel *et al.*, 1996). As can be seen from the packing diagram (Fig. 2), the crystal structure of (I) is stabilized by intermolecular N—H···O hydrogen bonding. The crystal packing is further stabilized by van der Waals forces

### Experimental

A solution of naphthalene disulfonyl chloride (384 mg, 1 mmol) dissolved in anhydrous CH_2_Cl_2_ (10 ml), and dropwise added over a period of 10 min to a solution of propan-2-amine (118 mg, 2 mmol) in CH_2_Cl_2_ (10 ml) at 273 K. The mixture was stirred at r.t. for 4 h. The organic phase was washed with water twice, and dried over anhydrous Na_2_SO_4_. The solvent was removed and the residue was purified by flash chromatography (1:3 cyclohexane:dichloromethane) to give ) as a white solid (267 mg, 62%). Single crystals suitable for X-ray measurements were obtained by recrystallization from ethanol and dichloromethane at room temperature.

### Refinement

H atoms were positioned geometrically and refined using a riding model, with C—H = 0.95–1.0 Å and N—H = 0.88 Å with *U*_iso_(H) = 1.2 (1.5 for methyl groups) times *U*_eq_(C,*N*).

### Crystal data

**Table:**

C_18_H_26_N_2_O_6_S_2_	*F*_000_ = 912
*M**_r_* = 430.53	*D*_x_ = 1.337 Mg m^−^^3^
Monoclinic, *P*2_1_/*c*	Mo *K*α radiation λ = 0.71073 Å
Hall symbol: -P 2ybc	Cell parameters from 875 reflections
*a* = 17.229 (3) Å	θ = 2.2–27.5º
*b* = 7.2532 (15) Å	µ = 0.28 mm^−^^1^
*c* = 18.035 (4) Å	*T* = 173 (2) K
β = 108.35 (3)º	Plate, colorless
*V* = 2139.2 (8) Å^3^	0.50 × 0.38 × 0.22 mm
*Z* = 4	

### Data collection

**Table:**

Rigaku R-AXIS RAPID IP area-detector diffractometer	4889 independent reflections
Radiation source: rotating anode	4227 reflections with *I* > 2σ(*I*)
Monochromator: graphite	*R*_int_ = 0.020
*T* = 173(2) K	θ_max_ = 27.5º
ω scans at fixed χ = 45°	θ_min_ = 1.3º
Absorption correction: multi-scan(ABSCOR; Higashi, 1995)	*h* = −22→22
*T*_min_ = 0.871, *T*_max_ = 0.940	*k* = −9→9
8740 measured reflections	*l* = −23→23

### Refinement

**Table:**

Refinement on *F*^2^	Secondary atom site location: difference Fourier map
Least-squares matrix: full	Hydrogen site location: inferred from neighbouring sites
*R*[*F*^2^ > 2σ(*F*^2^)] = 0.046	H-atom parameters constrained
*wR*(*F*^2^) = 0.117	*w* = 1/[σ^2^(*F*_o_^2^) + (0.0534*P*)^2^ + 0.9298*P*] where *P* = (*F*_o_^2^ + 2*F*_c_^2^)/3
*S* = 1.16	(Δ/σ)_max_ < 0.001
4889 reflections	Δρ_max_ = 0.36 e Å^−^^3^
253 parameters	Δρ_min_ = −0.49 e Å^−^^3^
Primary atom site location: structure-invariant direct methods	Extinction correction: none

### Special details

**Table:**

Geometry. All e.s.d.'s (except the e.s.d. in the dihedral angle between two l.s. planes) are estimated using the full covariance matrix. The cell e.s.d.'s are taken into account individually in the estimation of e.s.d.'s in distances, angles and torsion angles; correlations between e.s.d.'s in cell parameters are only used when they are defined by crystal symmetry. An approximate (isotropic) treatment of cell e.s.d.'s is used for estimating e.s.d.'s involving l.s. planes.
Refinement. Refinement of *F*^2^ against ALL reflections. The weighted *R*-factor *wR* and goodness of fit *S* are based on *F*^2^, conventional *R*-factors *R* are based on *F*, with *F* set to zero for negative *F*^2^. The threshold expression of *F*^2^ > σ(*F*^2^) is used only for calculating *R*-factors(gt) *etc*. and is not relevant to the choice of reflections for refinement. *R*-factors based on *F*^2^ are statistically about twice as large as those based on *F*, and *R*- factors based on ALL data will be even larger.

### Fractional atomic coordinates and isotropic or equivalent isotropic displacement parameters (Å^2^)

**Table:**

	*x*	*y*	*z*	*U*_iso_*/*U*_eq_	
S1	0.39879 (3)	0.72730 (7)	0.23076 (3)	0.02292 (13)	
S2	0.08210 (3)	1.11117 (7)	0.42543 (3)	0.01969 (13)	
O1	0.42832 (10)	0.4396 (2)	0.35142 (10)	0.0368 (4)	
O2	0.48381 (9)	0.7597 (2)	0.27099 (10)	0.0365 (4)	
O3	0.35149 (10)	0.8610 (2)	0.17682 (9)	0.0325 (4)	
O4	0.08620 (9)	1.2491 (2)	0.36997 (9)	0.0277 (3)	
O5	0.09471 (9)	1.1659 (2)	0.50512 (8)	0.0261 (3)	
O6	0.12159 (9)	0.7784 (2)	0.52066 (8)	0.0268 (3)	
N1	0.39535 (10)	0.5367 (2)	0.18585 (10)	0.0232 (4)	
H1A	0.4415	0.4763	0.1933	0.028*	
N2	−0.00546 (10)	1.0136 (2)	0.39554 (9)	0.0223 (4)	
H2B	−0.0358	1.0076	0.4266	0.027*	
C1	0.33009 (13)	0.5414 (3)	0.41377 (13)	0.0280 (5)	
H1B	0.3424	0.4413	0.4496	0.034*	
C2	0.37011 (13)	0.5567 (3)	0.35894 (13)	0.0262 (4)	
C3	0.35006 (12)	0.7046 (3)	0.30358 (11)	0.0212 (4)	
C4	0.29442 (12)	0.8349 (3)	0.30760 (11)	0.0209 (4)	
H4B	0.2823	0.9338	0.2712	0.025*	
C5	0.25444 (11)	0.8252 (3)	0.36502 (11)	0.0197 (4)	
C6	0.19712 (11)	0.9599 (3)	0.37008 (11)	0.0197 (4)	
H6A	0.1874	1.0640	0.3365	0.024*	
C7	0.15537 (11)	0.9418 (3)	0.42305 (11)	0.0190 (4)	
C8	0.16906 (12)	0.7855 (3)	0.47337 (11)	0.0215 (4)	
C9	0.22660 (13)	0.6566 (3)	0.47142 (12)	0.0247 (4)	
H9A	0.2370	0.5553	0.5066	0.030*	
C10	0.27065 (12)	0.6734 (3)	0.41739 (11)	0.0222 (4)	
C11	0.4475 (2)	0.2809 (4)	0.40126 (18)	0.0543 (8)	
H11A	0.4905	0.2093	0.3897	0.081*	
H11B	0.3985	0.2044	0.3922	0.081*	
H11C	0.4666	0.3207	0.4560	0.081*	
C12	0.32048 (14)	0.4553 (3)	0.13290 (14)	0.0336 (5)	
H12A	0.2781	0.5540	0.1156	0.040*	
C13	0.28821 (17)	0.3054 (4)	0.1736 (2)	0.0527 (8)	
H13A	0.2766	0.3573	0.2191	0.079*	
H13B	0.3292	0.2075	0.1905	0.079*	
H13C	0.2379	0.2541	0.1374	0.079*	
C14	0.3397 (2)	0.3816 (5)	0.06177 (16)	0.0605 (9)	
H14A	0.3603	0.4820	0.0368	0.091*	
H14B	0.2899	0.3305	0.0246	0.091*	
H14C	0.3813	0.2847	0.0780	0.091*	
C15	0.12852 (15)	0.6187 (3)	0.56924 (14)	0.0333 (5)	
H15A	0.0910	0.6304	0.6001	0.050*	
H15B	0.1848	0.6082	0.6044	0.050*	
H15C	0.1145	0.5083	0.5364	0.050*	
C16	−0.03619 (12)	0.9332 (3)	0.31603 (11)	0.0237 (4)	
H16A	0.0085	0.9406	0.2917	0.028*	
C17	−0.10837 (15)	1.0449 (4)	0.26648 (13)	0.0365 (6)	
H17A	−0.0915	1.1732	0.2642	0.055*	
H17B	−0.1527	1.0400	0.2897	0.055*	
H17C	−0.1276	0.9935	0.2135	0.055*	
C18	−0.05611 (15)	0.7314 (3)	0.32295 (13)	0.0315 (5)	
H18A	−0.0071	0.6671	0.3553	0.047*	
H18B	−0.0746	0.6756	0.2708	0.047*	
H18C	−0.0995	0.7212	0.3472	0.047*	

### Atomic displacement parameters (Å^2^)

**Table:**

	*U*^11^	*U*^22^	*U*^33^	*U*^12^	*U*^13^	*U*^23^
S1	0.0250 (3)	0.0202 (3)	0.0293 (3)	−0.00251 (19)	0.0167 (2)	−0.0004 (2)
S2	0.0251 (3)	0.0173 (2)	0.0197 (2)	0.00184 (18)	0.01134 (18)	−0.00043 (19)
O1	0.0400 (9)	0.0387 (10)	0.0408 (9)	0.0213 (8)	0.0256 (8)	0.0147 (8)
O2	0.0269 (8)	0.0371 (10)	0.0497 (10)	−0.0128 (7)	0.0179 (7)	−0.0073 (8)
O3	0.0468 (10)	0.0232 (8)	0.0372 (9)	0.0067 (7)	0.0270 (8)	0.0073 (7)
O4	0.0373 (8)	0.0209 (8)	0.0307 (8)	0.0051 (6)	0.0192 (7)	0.0061 (6)
O5	0.0350 (8)	0.0238 (8)	0.0229 (7)	−0.0010 (6)	0.0143 (6)	−0.0054 (6)
O6	0.0377 (9)	0.0218 (8)	0.0297 (8)	0.0051 (6)	0.0232 (7)	0.0056 (6)
N1	0.0204 (8)	0.0233 (9)	0.0278 (9)	0.0050 (7)	0.0100 (7)	−0.0020 (7)
N2	0.0211 (8)	0.0296 (10)	0.0197 (8)	0.0004 (7)	0.0114 (7)	−0.0028 (7)
C1	0.0325 (12)	0.0280 (12)	0.0270 (10)	0.0091 (9)	0.0144 (9)	0.0078 (9)
C2	0.0250 (10)	0.0278 (11)	0.0284 (10)	0.0074 (8)	0.0120 (8)	0.0032 (9)
C3	0.0222 (10)	0.0210 (10)	0.0223 (9)	−0.0023 (8)	0.0099 (8)	−0.0008 (8)
C4	0.0227 (10)	0.0199 (10)	0.0210 (9)	−0.0019 (8)	0.0084 (8)	−0.0012 (8)
C5	0.0197 (9)	0.0207 (10)	0.0191 (9)	−0.0006 (7)	0.0069 (7)	−0.0003 (8)
C6	0.0218 (10)	0.0195 (10)	0.0187 (9)	−0.0003 (7)	0.0076 (7)	0.0015 (8)
C7	0.0208 (9)	0.0173 (9)	0.0193 (9)	0.0009 (7)	0.0070 (7)	−0.0012 (7)
C8	0.0256 (10)	0.0227 (10)	0.0189 (9)	−0.0004 (8)	0.0108 (8)	−0.0001 (8)
C9	0.0302 (11)	0.0229 (11)	0.0239 (10)	0.0047 (8)	0.0126 (9)	0.0048 (8)
C10	0.0233 (10)	0.0232 (10)	0.0211 (9)	0.0012 (8)	0.0085 (8)	−0.0003 (8)
C11	0.0676 (19)	0.0501 (17)	0.0593 (17)	0.0394 (15)	0.0400 (15)	0.0280 (14)
C12	0.0292 (12)	0.0283 (12)	0.0356 (12)	0.0092 (9)	−0.0008 (9)	−0.0077 (10)
C13	0.0341 (14)	0.0359 (15)	0.085 (2)	−0.0089 (11)	0.0150 (14)	−0.0131 (15)
C14	0.066 (2)	0.068 (2)	0.0356 (14)	0.0236 (17)	0.0000 (14)	−0.0182 (15)
C15	0.0495 (14)	0.0233 (11)	0.0372 (12)	0.0038 (10)	0.0281 (11)	0.0065 (10)
C16	0.0251 (10)	0.0297 (11)	0.0182 (9)	0.0022 (8)	0.0097 (8)	−0.0024 (8)
C17	0.0359 (13)	0.0399 (14)	0.0288 (11)	0.0101 (10)	0.0031 (10)	0.0007 (10)
C18	0.0407 (13)	0.0282 (12)	0.0264 (11)	−0.0013 (10)	0.0119 (10)	−0.0041 (9)

### Geometric parameters (Å, °)

**Table:**

S1—O3	1.4324 (16)	C8—C9	1.371 (3)
S1—O2	1.4345 (17)	C9—C10	1.416 (3)
S1—N1	1.5937 (18)	C9—H9A	0.9500
S1—C3	1.775 (2)	C11—H11A	0.9800
S2—O4	1.4320 (15)	C11—H11B	0.9800
S2—O5	1.4402 (15)	C11—H11C	0.9800
S2—N2	1.5984 (18)	C12—C13	1.512 (4)
S2—C7	1.772 (2)	C12—C14	1.520 (4)
O1—C2	1.353 (2)	C12—H12A	1.0000
O1—C11	1.434 (3)	C13—H13A	0.9800
O6—C8	1.356 (2)	C13—H13B	0.9800
O6—C15	1.434 (3)	C13—H13C	0.9800
N1—C12	1.466 (3)	C14—H14A	0.9800
N1—H1A	0.8800	C14—H14B	0.9800
N2—C16	1.483 (2)	C14—H14C	0.9800
N2—H2B	0.8800	C15—H15A	0.9800
C1—C2	1.377 (3)	C15—H15B	0.9800
C1—C10	1.418 (3)	C15—H15C	0.9800
C1—H1B	0.9500	C16—C17	1.517 (3)
C2—C3	1.432 (3)	C16—C18	1.517 (3)
C3—C4	1.364 (3)	C16—H16A	1.0000
C4—C5	1.415 (3)	C17—H17A	0.9800
C4—H4B	0.9500	C17—H17B	0.9800
C5—C6	1.412 (3)	C17—H17C	0.9800
C5—C10	1.420 (3)	C18—H18A	0.9800
C6—C7	1.371 (3)	C18—H18B	0.9800
C6—H6A	0.9500	C18—H18C	0.9800
C7—C8	1.424 (3)		
			
O3—S1—O2	120.31 (10)	C1—C10—C5	119.10 (18)
O3—S1—N1	108.65 (10)	O1—C11—H11A	109.5
O2—S1—N1	105.52 (10)	O1—C11—H11B	109.5
O3—S1—C3	105.36 (9)	H11A—C11—H11B	109.5
O2—S1—C3	106.66 (10)	O1—C11—H11C	109.5
N1—S1—C3	110.17 (9)	H11A—C11—H11C	109.5
O4—S2—O5	118.63 (9)	H11B—C11—H11C	109.5
O4—S2—N2	108.82 (10)	N1—C12—C13	110.9 (2)
O5—S2—N2	106.76 (9)	N1—C12—C14	108.1 (2)
O4—S2—C7	105.99 (9)	C13—C12—C14	111.5 (2)
O5—S2—C7	109.31 (9)	N1—C12—H12A	108.8
N2—S2—C7	106.79 (9)	C13—C12—H12A	108.8
C2—O1—C11	118.22 (18)	C14—C12—H12A	108.8
C8—O6—C15	117.67 (16)	C12—C13—H13A	109.5
C12—N1—S1	124.38 (14)	C12—C13—H13B	109.5
C12—N1—H1A	117.8	H13A—C13—H13B	109.5
S1—N1—H1A	117.8	C12—C13—H13C	109.5
C16—N2—S2	120.79 (13)	H13A—C13—H13C	109.5
C16—N2—H2B	119.6	H13B—C13—H13C	109.5
S2—N2—H2B	119.6	C12—C14—H14A	109.5
C2—C1—C10	120.7 (2)	C12—C14—H14B	109.5
C2—C1—H1B	119.6	H14A—C14—H14B	109.5
C10—C1—H1B	119.6	C12—C14—H14C	109.5
O1—C2—C1	125.2 (2)	H14A—C14—H14C	109.5
O1—C2—C3	115.08 (18)	H14B—C14—H14C	109.5
C1—C2—C3	119.73 (19)	O6—C15—H15A	109.5
C4—C3—C2	120.08 (18)	O6—C15—H15B	109.5
C4—C3—S1	118.62 (15)	H15A—C15—H15B	109.5
C2—C3—S1	121.27 (15)	O6—C15—H15C	109.5
C3—C4—C5	121.11 (19)	H15A—C15—H15C	109.5
C3—C4—H4B	119.4	H15B—C15—H15C	109.5
C5—C4—H4B	119.4	N2—C16—C17	109.64 (17)
C6—C5—C4	121.62 (18)	N2—C16—C18	108.73 (17)
C6—C5—C10	119.25 (17)	C17—C16—C18	113.51 (19)
C4—C5—C10	119.10 (18)	N2—C16—H16A	108.3
C7—C6—C5	120.63 (18)	C17—C16—H16A	108.3
C7—C6—H6A	119.7	C18—C16—H16A	108.3
C5—C6—H6A	119.7	C16—C17—H17A	109.5
C6—C7—C8	120.15 (18)	C16—C17—H17B	109.5
C6—C7—S2	118.97 (15)	H17A—C17—H17B	109.5
C8—C7—S2	120.84 (14)	C16—C17—H17C	109.5
O6—C8—C9	125.04 (18)	H17A—C17—H17C	109.5
O6—C8—C7	114.81 (17)	H17B—C17—H17C	109.5
C9—C8—C7	120.14 (18)	C16—C18—H18A	109.5
C8—C9—C10	120.50 (19)	C16—C18—H18B	109.5
C8—C9—H9A	119.8	H18A—C18—H18B	109.5
C10—C9—H9A	119.8	C16—C18—H18C	109.5
C9—C10—C1	121.66 (19)	H18A—C18—H18C	109.5
C9—C10—C5	119.23 (18)	H18B—C18—H18C	109.5
			
O3—S1—N1—C12	−45.7 (2)	C5—C6—C7—S2	−178.34 (15)
O2—S1—N1—C12	−175.98 (18)	O4—S2—C7—C6	−3.88 (19)
C3—S1—N1—C12	69.3 (2)	O5—S2—C7—C6	−132.83 (16)
O4—S2—N2—C16	53.43 (18)	N2—S2—C7—C6	112.02 (16)
O5—S2—N2—C16	−177.42 (15)	O4—S2—C7—C8	178.22 (16)
C7—S2—N2—C16	−60.57 (18)	O5—S2—C7—C8	49.27 (18)
C11—O1—C2—C1	3.5 (4)	N2—S2—C7—C8	−65.88 (18)
C11—O1—C2—C3	−175.7 (2)	C15—O6—C8—C9	−3.8 (3)
C10—C1—C2—O1	179.1 (2)	C15—O6—C8—C7	176.08 (18)
C10—C1—C2—C3	−1.7 (3)	C6—C7—C8—O6	−177.07 (18)
O1—C2—C3—C4	−177.49 (19)	S2—C7—C8—O6	0.8 (2)
C1—C2—C3—C4	3.2 (3)	C6—C7—C8—C9	2.8 (3)
O1—C2—C3—S1	0.4 (3)	S2—C7—C8—C9	−179.31 (16)
C1—C2—C3—S1	−178.88 (18)	O6—C8—C9—C10	177.37 (19)
O3—S1—C3—C4	−14.70 (19)	C7—C8—C9—C10	−2.5 (3)
O2—S1—C3—C4	114.23 (17)	C8—C9—C10—C1	−179.6 (2)
N1—S1—C3—C4	−131.72 (16)	C8—C9—C10—C5	−0.1 (3)
O3—S1—C3—C2	167.35 (17)	C2—C1—C10—C9	177.6 (2)
O2—S1—C3—C2	−63.72 (19)	C2—C1—C10—C5	−1.8 (3)
N1—S1—C3—C2	50.3 (2)	C6—C5—C10—C9	2.5 (3)
C2—C3—C4—C5	−1.2 (3)	C4—C5—C10—C9	−175.71 (19)
S1—C3—C4—C5	−179.20 (15)	C6—C5—C10—C1	−178.05 (19)
C3—C4—C5—C6	179.59 (19)	C4—C5—C10—C1	3.7 (3)
C3—C4—C5—C10	−2.2 (3)	S1—N1—C12—C13	−100.2 (2)
C4—C5—C6—C7	175.95 (18)	S1—N1—C12—C14	137.2 (2)
C10—C5—C6—C7	−2.2 (3)	S2—N2—C16—C17	−111.90 (18)
C5—C6—C7—C8	−0.4 (3)	S2—N2—C16—C18	123.47 (17)

### Hydrogen-bond geometry (Å, °)

**Table:**

*D*—H···*A*	*D*—H	H···*A*	*D*···*A*	*D*—H···*A*
N1—H1A···O2^i^	0.88	2.00	2.821 (2)	154
N2—H2B···O5^ii^	0.88	2.22	3.001 (2)	148
N2—H2B···O6^ii^	0.88	2.53	3.235 (2)	138

Symmetry codes: (i) −*x*+1, *y*−1/2, −*z*+1/2; (ii) −*x*, −*y*+2, −*z*+1.
